# Treatment of Danish Survivors of Child Sexual Abuse—A Cohort Study

**DOI:** 10.3390/bs5040589

**Published:** 2015-12-11

**Authors:** Ask Elklit

**Affiliations:** Department of Psychology, University of Southern Denmark, Campusvej 55, DK-5230 Odense M, Denmark; E-Mail: aelklit@health.sdu.dk; Tel.: +45-6550-2320

**Keywords:** Treatment, adults, child sexual abuse, posttraumatic stress disorder, attachment, coping

## Abstract

Objective: To investigate the changes in psychological and social domains associated with treatment in survivors of child sexual abuse. Method: Participants from four centers were assessed at baseline and were followed up after six and 12 months. The battery covered posttraumatic and general distress symptoms, attachment, coping styles, self-worth, and social support. Results: The estimated prevalence of Posttraumatic Stress Disorder (PTSD) was 78% at baseline; this rate declined to 40% after one year. There were no differences in outcome measures across the different centers or between the individual and group treatments. Half of the PTSD variation at 12 months was explained by four factors: education, avoidance attachment, emotional coping, and social support. Conclusion: The findings in this study indicated a substantial reduction in mental health problems in survivors following 12 months of treatment and identified personality and social factors important for recovery.

## 1. Introduction

Child sexual abuse (CSA) has been widely recognized as the cause of much suffering in the lives of the adult survivors. Prior research has provided strong evidence that CSA is associated with a number of psychological, social, and physical health problems. Posttraumatic stress disorder (PTSD), depression, anxiety, somatoform disorder, alcohol and drug abuse, and borderline symptomatology are common disorders among adult CSA survivors [1,2,3,4,5,6]. Other studies have found that the consequences include sexual disorders [7,8], sexual risk behavior [9], revictimization [10], and intimate partner violence [11]. Recently, it has been found that many physical health disorders are related to CSA [12,13]. In addition, CSA survivors have been found to achieve less in education and income [14].

Because of the multiple devastating effects of CSA, there has been a growing interest during the last decades, in finding a treatment that can alleviate the suffering of the CSA survivors. A recent review by Taylor and Harvey [15] based on 44 studies concluded that psychological treatment of CSA had in general a predominantly moderate effect on most of the outcome domains. The effect sizes were in the range of 0.40–0.77 for PTSD, externalizing and internalizing symptoms, self-esteem, and global functioning or symptoms. A number of limitations were presented in the review. Many of the studies had a modest sample size (ranging from three to 198), only seven studies had more than 100 participants, only five studies included men, and seven studies included types of child abuse other than CSA. Only a small number of the studies lasted for more than 20 weeks or sessions and few studies reported follow-up results. In addition, there was a large variety of treatment modalities in the 44 studies, with only six studies based on cognitive behavioral or processing therapy. Despite these limitations, there was a remarkable consistency in overall effect sizes across the study designs.

Judith Herman [16] described a treatment strategy for CSA survivors, building on three phases; stabilization, trauma processing and recovery. A phase-oriented treatment acknowledges the vulnerabilities that characterize many of the survivors who have problems with trust and difficulties in emotional regulation. This step based treatment is also central to the “Skills Training in Affective and Interpersonal Regulation (STAIR)” treatment developed by Cloitre and Koenen [17], where interpersonal and emotional skills are trained before exposure begins.

Treatment for CSA survivors has recently become a concern for the government in Denmark and three regional treatment centers have been established. Each treatment center collaborates closely with centers that are run by volunteers. The treatment is carried out by psychologists under supervision. All of the centers complete a thorough assessment before treatment begins and repeat it every six months. There is no limit to the number of sessions and the treatment is free. All of the survivors receive weekly therapy; most of them on an individual basis. There is no common treatment manual. However, all of the centers use the personality oriented approach, based on Theodore Millon’s works [18] in the planning of the therapy. The most established center has published their treatment guidelines, which have built upon Judith Herman’s work. This treatment is a personality-guided approach, which focuses on emotional regulation, relationship issues and several other treatment modalities [19]. The client will typically stay in treatment for about 1½ years and as the therapy progresses, it may be relevant to make changes to the treatment modalities. This study examined the effects of the treatment within the new centers. Some of the treatment centers existed before the present structure. We decided to incorporate the treatment data that originated from before the new structure was established. One former center ran only treatment groups, while the other centers ran groups occasionally.

CSA has devastating effects on so many areas of life. Besides studying possible changes in trauma symptoms it is also important, to investigate other psychological variables that may have been impaired, as they may be central to the recovery process. Attachment orientation is established early in life as an instinctive reaction that can protect the infant in the case of danger. It has potential survival value and is common in mammals and birds. This orientation system is built on interpersonal experiences (including traumatic experiences) and it can be modified by experiences throughout the lifespan. It consists of two dimensions: attachment anxiety (the degree to which you trust someone for protection) and attachment avoidance (the degree of dependency and closeness you can tolerate). In the case of a life-threatening situation, attachment behaviors can evoke help or impede someone from getting help. In a toxic environment, the so-called maladaptive (e.g., insecure attachment styles) attachment orientation may have survival value. CSA or a dysfunctional family structure is likely to impair the attachment process resulting in insecure attachment. Hanna [20] found that adult female CSA survivors rated significantly higher on fearful attachment when compared to non-abused women. Cooper [21] found that significantly more of those with a history of CSA were characterized by a dismissive attachment style, in a sample of 245 female university students. Elklit [22] found that more than half of the CSA survivors had a fearful attachment style and only about 10% had a secure attachment style. The essence of attachment is to have a secure base when danger arises; the child will seek the help of protective parents or other adults nearby. Therefore, social relationships and the perception of social support are closely connected to attachment security.

Schumm, Briggs-Phillips, and Hobfoll [23] studied the association between perceived social support and PTSD among female victims of CSA. The study found that negative perceptions of current social support increased the women’s levels of PTSD symptomatology. A recent study by Stevens *et al.* [24] found that childhood maltreatment survivors, including CSA victims, reported low satisfaction with current social support. This lack of social support, among other variables, accounted for 63% of the variance in the symptoms of PTSD in the sample. Social support involves exchanges of information including emotional support and material resources. One’s ability to give and receive social support depends, partly on their feelings of self-worth. Social support has often been considered as a mediating variable. In this study, we have used it as an outcome measure because it was expected that the building of trust in the therapeutic relationship would be related to a major change in the clients’ perception of others.

Self-worth encompasses the extent to which individuals feel comfortable with their sense of self and also, to a lesser extent, their accomplishments, and how they believe they are viewed by others. A number of studies have demonstrated that CSA can have a negative impact on self-esteem. [25]. Romans *et al.* [26] found a clear relationship between poor self-esteem in adulthood and CSA, in participants that reported the more intrusive forms of abuse involving penetration. The most affected aspects of self-esteem were the increased expectation of unpleasant events and a sense of inability to influence external events whereas a sense of being attractive, having determination, or being able to relate to others were much less affected.

The coping strategies employed by survivors can also explain some of the variation in long-term functioning. Survivors who are able to effectively manage their negative emotions relating to the abuse are likely to experience less long-term distress than survivors who struggle to control these emotions. Coping methods have been categorized, as effective (e.g., directly addressing a problem) or ineffective (e.g., avoidance). The effectiveness of certain methods depends on the characteristics of the abuse and time since the abuse ended [27,28]. The above evidence has demonstrated that coping strategies can impact adjustment and functioning [29], therefore we would expect that variability in coping strategies could be used as an important indicator of the emotional and behavioral changes associated with CSA. In this study coping method was treated as outcome measure.

In this study, CSA was defined as incest, in which the abusive act was perpetrated by a family member. The term family was understood in a broad sense, as it also included close adult friends of the family. If the age difference between the victim and the perpetrator exceeded five years, we classed the sexual experiences as abuse, even if the interaction had been consensual. Of course, a child could also be sexually abused by a stranger, but the family would usually be able to protect and help the child at disclosure.

The present study aimed to investigate changes in mental health status during 12 months of therapy for CSA survivors and identify factors that can explain the variation of PTSD severity. We expected that there would be a reduction in PTSD and distress symptoms and also positive changes in several psychological domains. We would also like to identify factors that can predict PTSD severity after 12 months because such findings could inform the planning of therapy.

## 2. Method

### 2.1. Participants

Women and men eligible for the study were all consecutive outpatients (*n* = 480) at four (now three) regional treatment centers in Denmark that exist for individuals who were sexually abused in childhood. Exclusion criteria were (a) an active alcohol or drug misuse; (b) psychotic state; (c) massive self-destructive behavior; (d) current treatment elsewhere; and (e) a personality disorder with dominant perpetrating traits. Excluded clients were referred to either specialized institutions or to the affiliated volunteer centers. A small group of clients withdrew from the service within the first month but following this period most clients were stable attendees. A number of therapies were halted due to hospitalization or other serious life events. Regretfully, we were unable to get the exact attrition numbers. The recruitment rate was 100% (*n* = 480) due to the fact that the assessment was an integrated part of the treatment planning and monitoring of the centers. Most of the participants were women (85%) and almost all of the participants (91%) had experienced CSA before the age of 15, committed by a person at least five years older. The mean age of the sample was 36.4 years (SD = 10.8; range 15 to 70 years) and all participants were Caucasian. Fifty-one percent were married or cohabiting. The average length of education was 13.3 years (SD = 3.3; range 7 to 24 years). Almost two-thirds of the participants (59%) had children.

### 2.2. Procedure

All participants were informed that they would be asked to fill out a number of questionnaires during their second session (T1), which were used to guide the therapeutic process. The assessments were repeated at intervals of six and twelve months (T2 and T3). The study was approved by the Danish Data Agency and the IRB of the University of Southern Denmark.

### 2.3. Measures

Participants were first asked questions about their abuse history, such as onset, duration, relationship to the perpetrator, legal consequences for the perpetrator, and the time of abuse disclosure. Types of abuse were classified into 3 types: non-contact (spoken to about sexual matters, questioned about sexuality, teased about sexual development, had to listen to others’ sexual experiences, proposals or threats about taking part in sexual acts, made to watch someone present their genitals, watch adult intercourse or pornographic material, and present own genitals to someone else), non-penetrative contact (Kissed or fondled in a sexual way, touched area other than genitals in a sexual way, genitals were touched in a sexual way, had to touch or fondle the genitals of someone else, had to masturbate while someone was watching, and reciprocal masturbation), and penetrative contact (attempted intercourse, oral intercourse, anal intercourse, and genital intercourse). These questions were answered “Yes” or “No”. Descriptions of sexual victimization that did not fit into the respective items of the questionnaire regarding types of sexual abuse were registered in the “other types of sexual assaults” item (Table 1).

**Table 1:** Perpetrator and abusive acts, count and percentages.

Perpetrator Details		Abusive Acts	
*Person*	*Numbers*	*Activity*	*Numbers*
Mother	7 (5%)	Sexual talk	71 (53%)
Father	40 (30%)	Sexual questioning	38 (29%)
Stepparent	18(14%)	Sexual teasing	46 (35%)
Other family member	35 (26%)	Sexual suggestions	58 (44%)
Other adult	39 (29%)	Sexual exposure	88 (66%)
Sibling	15 (11%)	Pornography	30 (32%)
Several perpetrators	18 (14%)	Self-exposure	62 (47%)
Age difference > 5 years	113 (90%)	Sexual touched	102 (77%)
		Touch of genitals	75 (56%)
		Masturbation someone watch	15 (21%)
		Masturbation reciprocal	22 (17%)
		Coitus attempts	48 (36%)
		Oral penetration	34 (26%)
		Anal penetration	21 (16%)
		Vaginal penetration	30 (23%)
		Other forms of sex	23 (17%)
		Mean number of abusive acts (SD)	7.67 (4.31)

The Harvard Trauma Questionnaire part IV (HTQ) [30] was applied for estimating the occurrence of PTSD at the time of the present study. It consists of 30 items, 16 of which correspond to PTSD symptoms in the DSM-IV [31]. Participants who are just one avoidance or arousal symptom short of a full diagnosis are considered to have subclinical PTSD. Besides using the DSM-IV algorithm for the diagnosis, we also used the HTQ total score as a measure for PTSD severity. Mollica *et al.* [30] found good reliability and criterion validity for the HTQ. The HTQ-Part IV has been validated and used extensively in Denmark [32]. A sample item reads: “Are you on guard?”

The Trauma Symptom Checklist (TSC) was originally created by Briere and Runtz [33] as a measure of traumatic impact following CSA. The total score has been shown to be a valid measure of general psychological distress after a traumatic event, and was thus employed in the present study. A sample item reads: “Do you have problems in breathing?”

The Revised Adult Attachment Scale (RAAS) [34,35] is based on attachment theory [36]. The scale consists of 18 items relating to how adult respondents act and feel in relationships with others. Items are scored on a 5-point Likert scale. An example item is: “I feel other people are more distant than I like”. The scale contains three subscales (closeness, dependency, and anxiety). The first two subscales are combined into an avoidance attachment dimension, where high numbers represent closeness and dependency. The scale has good reliability and validity [34].

The World Assumption Scale (WAS) [37] is a 32-item checklist of assumptions. Respondents are asked to indicate on a 6-point Likert Scale, ranging from “strongly disagree” to “strongly agree” the degree to which they consider a certain statement appropriate. Research suggests that the scale discriminates well on degrees of traumatization, and that it performs uniformly across cultures [38]. The self-worth subscale was considered to be the most relevant as it was expected that it would change throughout the course of the therapy (e.g., “I often think I am worth nothing”).

The Coping Styles Questionnaire (CSQ) [39] was used to measure coping strategies. CSQ in its original form includes 60 items, measuring four primary coping styles: rational, emotional, avoidance, and detached. All items are scored on a 4-point Likert scale. Elklit [40] made a validation study on Danish respondents and proposed a shorter version with 37 items. In this study, we only used the subscales of emotional coping (10 items) and avoidance coping (10 items) that were considered to be the most relevant in relation to therapeutic changes. Examples are “I blame myself” and “I take one step at time”.

The Crisis Support Scale (CSS) [41] was used to measure experience of perceived social support after a traumatic event. The scale comprises the following items: emotional support (two items), practical support, contact with people in a similar situation, the ability to express oneself, the experience of being let down, and general satisfaction with social support. Items are rated on a 7-point Likert Scale, ranging from “never” to “always”. The CSS has been used in many trauma studies and it has a good internal consistency as well as good discriminatory power. Elklit, Pedersen, and Jind [42] analyzed 4213 CSS questionnaires, from 11 studies, and the results confirmed the psychometric reliability and validity of the CSS.

The psychometric values of all the scales used in the current study are presented in Table 2.

**Table 2:** Changes in psychological variables during 12 months of treatment.

Variable	α	T1	T2	T3	T1–T2		T1–T3	
		Mean (SD)	Mean (SD)	Mean (SD)	T	df	T	df
HTQ total	0.93	85.5 (17.5)	74.8 (19.8)	71.2 (19.9)	9.6 *	232	11.4 *	114
TSC total	0.87	76.4 (17.0)	67.4 (20.6)	62.9 (19.2)	6.3 *	220	9.3 *	123
Emotional coping	0.85	24.9 (6.2)	22.3 (6.3)	22.0 (6.9)	7.5 *	255	8.6 *	139
Avoiding coping	0.65	21.4 (4.4)	20.2 (4.0)	20.3 (4.9)	4.4 *	258	1.9	141
Anxious attachment	0.78	20.4 (6.2)	18.7 (6.0)	18.5 (6.2)	4.7 *	263	5.6 *	143
Avoidant attachment	0.77	32.5 (8.4)	35.8 (9.2)	36.3 (9.1)	−5.5 *	261	−6.7 *	136
Self-worth	0.70	19.2 (5.0)	20.9 (5.1)	21.6 (5.4)	−6.7 *	272	−7.3 *	148
Social support	0.74	30.5 (7.4)	31.7 (14.3)	32.9 (8.3)	−1.4	273	−5.2 *	148

Note: HTQ = Harvard Trauma Questionnaire; TSC = Trauma Symptom Checklist; * *p* < 0.0005.

### 2.4. Statistics

Nominal variables were compared with χ^2^ tests. A 0/1 (no/yes) coding was used for dichotomous variables. Correlations were estimated with Pearson’s correlation coefficient. Demographic, treatment, and life event variables were analyzed in relation to all scales with one-way ANOVA. Given the wide variation in the age of participants, analyses were conducted that found that the time since abuse was not predictive of any outcome variables. To reduce the risk of Type 1 errors, only symptom total scores were used in subsequent analyses. T-tests were conducted to examine change over time in the socio-psychological variables. Effect size was calculated by Cohen’s δ. Hierarchical multiple regression analyses were performed to find variables that predicted PTSD severity 12 months later. The analyses were performed by means of SPSS-PC, version 20.0.

## 3. Results

### 3.1. Abuse Characteristics

Table 3 reports the descriptive statistics for the group that stayed in treatment for one year. No significant differences in demographics, perpetrators data, or abusive acts were found between the baseline group and the group that received one year of treatment. Table 1 reports details perpetrator status and what type of sexual acts the respondents were exposed to. The reported rates were not mutually exclusive and the clients could endorse more than one. In fact, most participants had experienced a considerable number of abusive acts (mean 7.7). This indicated that some children may grow up in a family where they are treated as a sexual object in many ways. The concept of “*grooming*” could be an adequate way of understanding the relationship between the many, different acts of abuse. We did not ask how often nor at what age the various acts happened. For some of the survivors, it was “a tough round” to answer this part of the assessment. Many, however, said that it also meant to them that the therapists knew “what it was all about” and immediately felt confidence in the quality of treatment. This part of the assessment was not repeated at T2 and T3.

**Table 3:** Descriptive statistics and test statistics for differences between T1 and T3 responders.

	Baseline Sample	One Year Sample	*F* Ratio/χ^2^
	Mean (SD)/%	Mean (SD)/%	
Age	36.4 (10.76)	38.7 (9.90)	7.32 (*p* < 0.01)
Years of education	13.3 (3.50)	13.8 (3.99)	4.90 (*p* < 0.05)
Gender (% female)	83.4%	73%	χ^2^ 13.48 (*p* < 0.0005)
Employed	54%	55%	χ^2^ 0.17 *ns*
Marriage/cohabitation	50.4%	52%	χ^2^ 0.60 *ns*
Children	58.2%	62%	3.94 (*p* < 0.05)
Age when abuse started	6.5 (7.1)	7.1 (3.90)	1.60 *ns*
Duration (months) of abuse	87.7 (86.83)	75.5 (69.82)	0.01 *ns*
Disclosure age	22.1 (11.21)	23.1 (11.71)	1.84 *ns*
Body injuries	16.6%	17%	0.01 *ns*
Report police	16.5%	18%	0.27 *ns*
Court trial	10.3%	12%	0.01 *ns*
Conviction	9.7%	12%	0.48 *ns*

Note: *ns* = non-significant.

### 3.2. Traumatic Symptoms and Psychological Distress

At T1, 78% of the CSA survivors met the three core criteria of the PTSD diagnosis and 16% met the criteria for a subclinical PTSD diagnosis (fulfilling two out of three criteria). At T2, the number was reduced to 52% with PTSD (23% subclinical PTSD) and at T3 the 40% had PTSD and 23% subclinical PTSD. The changes in psychological measures reported by the participants are displayed in Table 2. The table reveals that there was a steady decrease in symptoms from T1 to T3. Furthermore, there were significant reductions in the other outcome measures: the maladaptive coping strategies, anxious attachment and avoidant attachment, and increases in reported self-worth and social support. The effect size (Cohen’s delta) was large (0.87) for the trauma symptoms (HTQ) after one year (0.65 after six months). For the distress symptoms (TSC), the effect sizes were moderate: 0.45 after six months and 0.74 after one year.

### 3.3. Background and Treatment Factors

There were no significant differences in outcome measures for participants who received individual or individual and group treatment, nor were there any significant differences between the centers (Table 2).

### 3.4. Regression Analysis

To identify which of psychological factors were predictors of PTSD severity after one year, a hierarchical regression analysis was conducted. Due to sample size considerations, firstly we investigated demographic factors in a separate regression analysis. Years of education were the only significant factor. In separate analyses we entered the abuse variables from Table 1; none of which were significant predictors. Next, we studied the baseline psychological variables separately and identified three that predicted PTSD severity, namely avoidant attachment, emotional coping, and social support. Therefore, the final model included years of education as the first step and was followed by avoidance attachment as the second step. In the third step, emotional coping style was added and in fourth step the total score for social support was added. The reason for having attachment as the second step was due to the assumed early development of attachment. Emotional coping style was chosen as the third step because we thought of it as a semi-trait quality. Social support may change for the incest survivors and could vary widely across different situations, it was therefore added in the final step.

We also considered controlling for the initial PTSD level. However, 84% of the participants had either full or subclinical PTSD. This factor alone was so dominant and could explain 49% of the variation in PTSD level at 12 months, therefore it was left out the final model. All of the independent factors were measured at baseline and the dependent factor was the HTQ score at 12 months. The final model explained 48% of the variance in PTSD severity (Table 4). When social support was introduced to the model attachment avoidance was no longer significant.

**Table 4:** Hierarchical Multiple Regression Models for Predicting PTSD Severity after 12 months.

Variable	Model 1		Model 2		Model 3		Model 4	
	*B* (*SE*)	β	*B* (*SE*)	β	*B* (*SE*)	β	*B* (*SE*)	β
Intercept	91.56 (6.71)		116.01 (7.70)		69.67 (13.74)		90.07 (13.56)	
Education	−1.06 (0.47)	−0.33 *	−1.08 (0.43)	−0.22 *	−0.97	−0.20 *	−0.86 (0.37)	−0.18 *
Avoidance attachment			−0.97 (0.40)	−0.45 *	−0.56 (0.21)	−0.26 *	−0.33 (0.20)	−0.15
Emotional coping					1.24 (0.31)	0.37 *	1.18 (0.29)	0.35 *
Social support							−0.91 (0.21)	−0.33 *

Note: PTSD = Posttraumatic Stress Disorder. For Model 1, adj *R*^2^ is 0.10; for Model 2, adj. *R*^2^ is 0.29; for Model 3, adj *R*^2^ is 0.38 and for Model 4, adj. *R*^2^ is 0.48. All models are significant (for all, *F* > 11.92 and *p* ≤ 0.001).

## 4. Discussion

In this study, we found that 12 months of treatment had a strong effect on trauma symptoms and had moderate effects on the general distress and self-worth outcome measures. The possible PTSD cases reduced from 78% to 40%. Four variables explained almost half of the variation of PTSD symptoms. A long education, avoidance attachment, and social support were negatively associated with PTSD severity, while emotional coping style had a positive association with PTSD outcome.

The treatment in the regional centers appeared to be effective. The effects of the treatment during the second half of the year were less than in the first six months, as one could expect. However, it would be likely that there would still be a number of symptoms in clients that would have to be addressed even after one year of treatment. It is possible that a number of the clients left treatment during the first year due to improved psychological well-being; but it is also possible that some have left treatment due to lack of improvement. We do not have any data on attrition; this would be very valuable for future evaluations.

Educational level improved the effectiveness of our model’s ability to predict PTSD severity, which no other demographic variable was able to do. Higher educational level is, all things been equal—synonymous with more resources, knowledge, insight, and improved access to social networks and other resources in time of need. Individuals with a higher level of education are likely to benefit more from the treatment. Therefore, it is reasonable to expect that it would be strongly associated with a reduction of PTSD over time.

In line with previous studies [20,22], the clients had high scores of avoidant and anxious attachment. The combination of the two dimensions is equivalent to the fearful attachment style. Elklit [22] found that half of a similar sample was characterized by fearful attachment style. The final regression analysis showed that the avoidance attachment style was associated with PTSD after 12 months. High avoidance attachment at baseline was a fairly strong predictor for positive treatment outcome one year later. An avoidant attachment style in many cases may have functioned as a protective shield against being betrayed and let down again. A high level of avoidance attachment may reflect difficulties in trusting others in close relationships. Trust is essential for the working alliance in therapy. For many survivors, the reconstruction of basic trust in others is a long and difficult process. In the hands of a caring and competent therapist who gets to know the survivor well, this distrust can be changed into a stable and positive relationship, which could potentially be transferred to other relationships outside of the therapy room.

Emotional coping was positively associated with PTSD severity after one year. Emotional coping is generally considered maladaptive after trauma and it has found to be associated with negative outcomes. Folkman and Lazarus [29] mentioned that emotional coping can be adaptive in situations where there are no possibilities to act, for example, when the individual is in a state of helplessness. Typically, the young child is subjugated to the total control of the perpetrator. Due to the young age when the abuse starts, they often believe what the perpetrator tells them, including the terrible things that will happen if they ever reveal the abuse.

Social support turned out to be negatively associated with PTSD outcome after one year, therefore high levels of social support will be predictive of a later decrease in PTSD. In this study, we viewed social support as an outcome of the treatment, as it was expected that trust would build up within the therapeutic relationship. Social support has traditionally been viewed as something that occurs outside of therapy and in many studies it has been treated as a moderator or a mediator. Our finding is in line with two influential meta-analyses [43,44] on the protective function of social support in relation to PTSD.

Several factors did not have enough strength in the final regression analysis such as avoidant coping and anxious attachment. A possible explanation for these findings could be the result of their small effect sizes when compared to the effect sizes of emotional coping and avoidant attachment, respectively. Self-worth was another obvious candidate, which did not have enough strength. It had a relatively high effect size but it was not a strong independent predictor. This could be due to the logical relationship that self-worth has with avoidant attachment on one side and social support on the other side.

Another important issue was the limited predictive value of several traditional demographic variables, including gender, marital status, number of children, and employment. The same was true of the characteristics of the abuse, which did not appear to have much of an impact on trauma recovery. This finding is in line with previous studies [5]. From a treatment perspective, there is no useful objective measure for abuse exposure other than the subjective appraisal. For a child, sexual abuse is immediately or almost immediately associated with strong negative feelings, that have to be suppressed. Once the abuse starts, many escape through dissociation; their world shatters, there is no sanctuary, and no help from protective and strong adults. The child is constantly vigilant in an attempt to avoid further abuse. The threshold for psychological damage is low and once it has been crossed, the number of acts and other details about the abuse do not seem to matter for PTSD severity as a treatment outcome.

A number of limitations should be mentioned. This study was based on an assessment battery with the possibility of a biased recall and subjective report. Most of the analyses were based on the number of clients that stayed in treatment for 12 months. It is possible that the clients who did not stay in therapy could be different from the clients who continued to attend for a whole year. However, very few differences were found at baseline between the two groups. In addition, there was no control group. In principle, the changes that occurred following treatment could be attributed to other factors outside of the treatment. However, we think that the heavy investment in time and the emotion processing that occurred during therapy were at least partly accountable for the good results. Some of the measures used in the study have been criticized, e.g., Walsh *et al.* questioned the use of general coping questionnaires within CSA [28] and asked for a more sensitive measure, which can specify the process of coping as it occurs.

Despite the limitations, this study has expanded on previous studies [15,22], by using a large sample of consecutive clients who were recruited from several centers. The sample included males and females and both individual and group therapy as treatment modalities. In addition, the treatment was conducted over a long period of time and assessments were conducted at three time points. All of the above strengths give this study good external validity.

## 5. Conclusions

The present study informs policymakers and therapists that long-term treatment of incest survivors is very effective and can lead to considerable decreases in trauma and distress symptomatology. This is very encouraging given the dire toll that these survivors may pay otherwise. As CSA impacts many domains of living, we included several psychological variables. Three factors of treatment relevance attracted special attention; avoidance attachment, emotional coping style, and social support. These three factors deserve focused attention as a means of reducing the heavy load that these clients carry when they come to the clinic.
